# An Analysis of ATV and UTV-Related Injuries Pre- and Post-Enactment of West Virginia Senate Bill 690

**DOI:** 10.1016/j.focus.2025.100335

**Published:** 2025-03-18

**Authors:** Toni M. Rudisill, Jeffrey Beyon, Patrick C. Bonasso, James M. Bardes

**Affiliations:** 1 Department of Epidemiology and Biostatistics, West Virginia University, Morgantown, West Virginia; 2 Department of Medicine, West Virginia University, Morgantown, West Virginia; 3 Department of Surgery, West Virginia University, Morgantown, West Virginia

**Keywords:** Injury rates, health policy, legislation, all-terrain vehicles

## Abstract

**Introduction:**

All-terrain vehicles and utility task vehicles are associated with a high incidence of injuries, particularly in West Virginia, which has the highest all-terrain vehicle-related fatality rate in the U.S. In 2020, the enactment of the West Virginia Senate Bill 690 allowed all-terrain vehicles and utility task vehicles on public roads, raising concerns about potential increases in injury rates. This study aimed to evaluate the impact of Senate Bill 690 on trauma admissions related to all-terrain vehicle and utility task vehicle injuries at a Level I trauma center in West Virginia.

**Methods:**

A cross-sectional study was conducted, analyzing trauma admissions from January 1, 2017, to June 30, 2023. Data were sourced from medical records and external databases, including population estimates, unemployment rates, and gasoline prices. Negative binomial regression was used to analyze the relationship between the number of trauma admissions and the enactment of the law, adjusting for multiple confounding factors.

**Results:**

A total of 1,318 individuals were treated for all-terrain vehicle or utility task vehicle injuries. After the law's enactment, injury rates increased by 14% (incidence rate ratio =1.14, 95% CI= 1.01,1.29) for the overall population and by 24% (incidence rate ratio=1.24, 95% CI=1.03, 1.48) after adjusting for patient demographics and socioeconomic variables.

**Conclusions:**

The rate of all-terrain vehicle and utility task vehicle-related trauma admissions significantly increased after the passage of Senate Bill 690 in West Virginia. Given that all-terrain vehicle and utility task vehicle injuries are already disproportionately high in West Virginia compared to other states, these findings may suggest additional intervention or policy changes are needed to limit morbidity and mortality associated with these types of injuries.

## HIGHLIGHTS

• West Virginia has the highest all-terrain vehicle injury rate in the nation.
• Senate Bill 690 was passed in March 2020 allowing all-terrain vehicles on public roads.
• This study investigated the injury rates before and after Senate Bill 690’s passage.
• The injury rate increased 24% after adjusting for patient demographics and socioeconomic variables.

## INTRODUCTION

All-terrain vehicles (ATVs) and utility task vehicles (UTVs) are motorized vehicles designed for use in off-road working environments. Because they differ from traditional automobiles, especially in terms of safety requirements, ATV and UTV-related injuries continue to be a significant concern among the medical community. The U.S. Consumer Product Safety Commission (CPSC) reported more than 3,000 children aged younger than 16 years have died from such injuries between 1982 and 2015, with nearly a million more who visited the emergency department.^1^ However, recent reviews of all relevant literature describe consistent patient characteristics; most patients treated for ATV or UTV-related injuries tend to be male, aged younger than 18 years, rarely wore helmets at the time of injury, resided in more rural locations, and suffered orthopedic and/or head injuries.^1^, ^2^, ^3^, ^4^, ^5^, ^6^

To address this public health concern, several interventions have been proposed, including helmet use, public education, and legislation. Many states have legislation limiting the use of these vehicles on public roads. West Virginia was one such state; however, on March 7, 2020, the West Virginia Senate Bill 690 was passed to allow ATV and UTV riders to use public roads so long as certain stipulations were met.^7^ The bill required these vehicles to have similar features as automobiles, including head and tail lighting, reflectors, mirrors, windshields, a horn, and a speedometer. The legislation did not require additional safety measures such as lap belts or the use of helmets. If properly equipped, then ATVs and UTVs could be registered as street-legal special purpose vehicles.^7^ It should be noted that West Virginia's existing ATV/UTV law requires individuals aged under 18 years to wear a helmet when riding.

West Virginia has the highest fatality rate related to ATV usage compared to the rest of the nation.^8^ Because of this disparity, a significant body of epidemiologic literature exists regarding ATV-related injuries in West Virginia.^2^^,^^5^^,^^9^, ^10^, ^11^, ^12^, ^13^, ^14^, ^15^, ^16^, ^17^ Because the mortality and morbidity related to ATV and UTV usage is already elevated in West Virginia, it is unknown how allowing them on public roads impact population level injury rates. Previous research determined ATV collisions that occur on public roads tend to result in more fatalities than those that occur off-road.^18^^,^^19^ Previous studies have also shown more unsafe riding behaviors (e.g., speeding, lower helmet use, increased substance use) occur on paved versus unpaved roads.^20^^,^^21^

Several studies have examined the relationship between certain types or aspects of legislation and ATV/UTV-related injuries, such as age or helmet restrictions.^21^, ^22^, ^23^, ^24^, ^25^, ^26^, ^27^ However, only one study to date, which was conducted in Iowa, investigated the relationship between crash rates and the enactment of county-level policies that permitted off-road vehicles on public roads; this study found that crashes involving off-road vehicles on public roads increased 58% in counties that passed laws allowing these vehicles on public roads.^28^ To the authors’ knowledge, no study to date has investigated the relationship between trauma admissions and a statewide law that allows off-road vehicles on public roads. Thus, the purpose of this study was to evaluate the potential impact of West Virginia Senate Bill 690 on trauma admissions at one of West Virginia's adult Level I and only pediatric Level II trauma centers. The study aimed to evaluate changes in injury rates before and after the adoption of the bill. Based on the limited extant literature, it was hypothesized that the increase in street-legal vehicles would lead to an increase in ATV and UTV-related trauma admissions.^28^

## METHODS

### Study Population

This cross-sectional study included any individual treated at the Jon Michael Moore Trauma Center in Morgantown, West Virginia, for an ATV or UTV-related injury that occurred between January 1, 2017, and June 30, 2023. Patients were identified using ICD-10 codes. Codes included were V86.05XA, V86.09XA, V86.15XA, V86.19XA, V86.55XA, V86.59XA, V86.65XA, and V86.69X. This study was approved by our university's Institutional Review Board (protocol # 2397810349).

### Measures

Six separate data sources were used for this analysis. The first data source was the patients’ medical records. Data included the type of vehicle (e.g., ATV or UTV), patient demographics (e.g., age at injury, biological sex), vehicle position (e.g., driver or passenger), and helmet use at the time of injury (e.g., yes, no, unknown/missing). The second data source was West Virginia's population estimates as of July 1, which were obtained by year for the overall population, by age (eg, <18 years and ≥18 years), and by sex, from the U.S. Census Bureau.^29^

As economic factors can influence motor vehicle usage,^30^^,^^31^ the unemployment rate and gasoline prices were incorporated into the analyses. The third data source was the monthly unemployment rates for West Virginia, which were obtained from the U.S. Bureau of Labor Statistics.^32^ The fourth data source was the monthly, unadjusted retail gasoline prices, which were gathered from the Energy Information Administration.^33^ The fifth data source was the monthly consumer price index, which was obtained from the Federal Reserve.^34^

Lastly, as law enforcement may impact a population's compliance with traffic laws,^35^ the number of police and sheriffs’ patrol officers employed in West Virginia per year was obtained from the U.S. Bureau of Labor Statistics.^36^

The primary dependent variable in this study was the number of ATV or UTV trauma admissions. These counts were summed by quarter-year for the overall population and by age group (e.g., <18 years and ≥18 years) and by sex (female and male). This grouping decision was made based on existing literature, which shows ATV/UTV-related injuries can vary by age and sex.^37^ In addition, the quarter of the year is important because it reflects seasonality, which may influence ATV/UTV riding. Quarter 1 includes January-March, quarter 2 includes April-June, quarter 3 includes July-September, and quarter 4 includes October-December. The primary independent variable was whether Senate Bill 690 was in effect during that quarter-year. Although the law was passed on March 7, 2020, it went into effect 90 days later, on June 5, 2020. Thus, each quarter-year was binarily coded to designate whether the bill was or was not in effect (e.g., 1 = yes in effect; 0 = not in effect). All population estimates were linearly interpolated into quarter-year. The monthly unemployment rate was averaged for each quarter-year and then logged to achieve linearity. The number of patrol officers employed by year was linearly interpolated into quarter-year. The monthly retail gasoline prices were adjusted by the June 2023 consumer price index to correct for inflation; these values were then averaged for each quarter-year.

### Statistical Analysis

Descriptive statistics of the study population were assessed through percentages and frequencies. Before running analyses, all independent variables previously described along with quarter and year were assessed for collinearity using variance inflation factor in a linear regression model. It was found that the year heavily correlated with several variables and thus not included in any further models. Likewise, it was found that the number of police and adjusted gas price were also heavily correlated with one another and thus modelled separately. Because the primary dependent variable involved count data that were over-dispersed, negative binomial regression was used to assess the unadjusted and adjusted injury rates per capita.^38^ The population estimates were logged and used as the offset in the models.

The first set of models (N=5) assessed the relationship between the number of trauma admissions and the presence of the law for the overall population. The first model (Model 1) contained only the counts of trauma admissions and the presence/absence of the law. Model 2 adjusted for the variables in Model 1 plus the quarter. The third model (Model 3) contained those in Model 2 plus the log of unemployment. Model 4 contained all the variables in Model 3 plus the number of police. Model 5 contained all the variables in Model 3 plus the adjusted gasoline price. The models were structured this way to show how each additional variable impacted (i.e., increased or decreased) the injury rate.

The second set of models (N=5) assessed the relationship between the number of trauma admissions and the presence or absence of the law while adjusting for patients’ sex and age. Model 6 contained the number of trauma admissions, the presence or absence of the law, patients’ sex, and patients’ age group. Model 7 contained all the variables in Model 6 plus the quarter. Model 8 contained all the variables in Model 7 plus the log of unemployment. Model 9 included all the variables in Model 8 plus the number of police. The last model (Model 10) included all the variables in Model 8 plus the adjusted gas price. All data management and analyses were conducted using SAS/STAT software, version 9.4, with two-sided hypothesis testing and α=0.05.

## RESULTS

Between January 1, 2017, and June 30, 2023, 1,318 individuals were treated for ATV or UTV-related injuries (Table 1). Nearly 76% of treated patients were male, 79% drove ATVs compared to UTVs, and nearly 79% were drivers as opposed to being passengers. Helmet usage was very low (17%). In addition, more injuries occurred after Senate Bill 690 went into effect (55% vs 44% before the bill).

**Table 1:** Descriptive Characteristics of Patients Treated for ATV/UTV Injuries, January 1, 2017–June 30, 2023 (N=1,318)^a^

Characteristic	Frequency	Percent
Patient's age (years)		
<18	349	26.5
≥18	969	73.5
Sex		
Female	321	24.4
Male	997	75.6
Vehicle		
ATV	1,035	78.5
UTV with cage	283	21.5
Position		
Driver	1,040	78.9
Passenger	253	19.2
Unknown	25	1.9
Helmet Use		
Yes	224	17.0
No	838	63.6
Unknown	256	19.4
Senate Bill 690 in effect		
Yes	728	55.2
No	590	44.8

a Percentages may not add to 100% due to rounding. ATV, all-terrain vehicles; UTV,utility task vehicles.

After adjusting for law, quarter, unemployment rate, and gasoline prices, the injury rate for the overall population was 14% higher after the passage of the law (incident rate ratio=1.14, 95% CI=1.01, 1.29) (Table 2). Likewise, after adjusting for patients’ sex, age, law, quarter, unemployment, and gasoline prices, the injury rate was 24% higher after the passage of the law (incident rate ratio=1.24, 95% CI=1.03, 1.48) (Table 3). It should also be noted that when considering the law change, injuries were much greater among males and adults.

**Table 2:** ATV/UTV Injuries per Capita Requiring Emergency Medical Treatment After Passage of Senate Bill 690^a^

	Model 1	Model 2	Model 3	Model 4	Model 5
	IRR (95% CI)	IRR (95% CI)	IRR (95% CI)	IRR (95% CI)	IRR (95% CI)
Law in effect (Yes versus No)	1.25 (0.79, 1.98)	1.15 (1.01, 1.32)	1.14 (1.01, 1.29)	1.11 (0.94, 1.32)	1.14 (1.01, 1.29)

a Negative binomial regression was used to calculate the IRR in all models. The total West Virginia population was used as the offset. Each model contained the following variables: Model 1: Law only. Model 2: Law, quarter. Model 3: Law, quarter, log of unemployment. Model 4: Law, quarter, log of unemployment, number of police. Model 5: Law, quarter, log of unemployment, adjusted gas price. IRR, incidence rate ratio.

**Table 3:** ATV/UTV Injuries per Capita Requiring Emergency Medical Treatment After Passage of Senate Bill 690 by Sex and Age

	Model 6	Model 7	Model 8	Model 9	Model 10
	IR (95% CI)	IR (95% CI)	IR (95% CI)	IR (95% CI)	IR (95% CI)
Law in effect (Yes versus No)	1.35 (1.02, 1.78)	1.23 (1.03, 1.48)	1.23 (1.03, 1.47)	1.23 (0.97, 1.58)	1.24 (1.03, 1.48)
Sex (Male versus Female)	10.65 (8.04, 14.11)	11.71 (9.70, 14.13)	11.76 (9.76, 14.17)	11.76 (9.76, 14.17)	11.76 (9.76, 14.17)
Age (Adult versus Child)	2.43 (1.84, 3.21)	2.50 (2.08, 3.01)	2.50 (2.08, 3.00)	2.50 (2.08, 3.00)	2.50 (2.09, 3.01)

^a^Negative binomial regression was used to calculate the IRR in all models. For sex, females served as the referent. For age, children (i.e., those <18 years of age) served as the referent. The West Virginia population by age and sex were used as the offset. Each model contained the following variables:

Model 6: Law, sex, age.

Model 7: Law, sex, age, quarter.

Model 8: Law, sex, age, quarter, log of unemployment.

Model 9: Law, sex, age, quarter, log of unemployment, number of police Model 10: Law, sex, age, quarter, log of unemployment, adjusted gas price.

IRR, incidence rate ratio.

## DISCUSSION

This study found that the passage of West Virginia's Senate Bill 690 was associated with a 24% increased rate of trauma admissions at one of the state's two adult Level 1 trauma centers or pediatric Level 2 trauma centers after adjusting for population characteristics and socioeconomic variables. These findings coalesce with the limited extant literature and have public health implications.

West Virginia experiences a disproportionate number of injuries and fatalities related to ATV/UTV use.^8^ Previous studies conducted in West Virginia have also shown that many of the injuries/fatalities involve drivers not wearing helmets at the time of injury who are male, and aged over 18 years.^2^^,^^5^^,^^9^, ^10^, ^11^, ^12^, ^13^, ^14^, ^15^, ^16^, ^17^ Although the literature is limited, a previous study conducted in Iowa found that ATV crash rates were significantly higher in counties that allowed off-road vehicles on public roads compared to counties that did not.^28^ In addition, previous studies determined ATV collisions that occur on public roads tend to be more severe.^18^^,^^19^ Therefore, the findings of the present study are aligned with the extant literature.

From a public health perspective, interventions to reduce ATV and UTV-related injuries are desperately needed in West Virginia. The injuries are common and do create economic and societal burdens.^12^ Besides legislation, focused interventions and rider education may be viable options to limit morbidity and mortality associated with ATV/UTV use. However, interventions to reduce ATV/UTV-related injuries are largely absent from the literature. A previous study conducted with both children and adult ATV users in Arkansas showed that riders want educational materials that include action-oriented safety information that is clear, realistic, and visually appealing.^39^ Two other studies have developed educational interventions targeting high school and middle school students, respectively.^40^^,^^41^ The program developed for high school students was delivered by a trauma surgeon and involved a simulator and discussions concerning safe riding practices; this program was found to be associated with fewer trauma admissions.^40^ The interventional program developed for middle school students was in Alabama and delivered by medical residents; the program focused on educating the students about safe riding practices using materials developed by the University of Iowa.^41^ This program was also found to be effective in increasing safe riding practices.^41^ However, given that ATV injuries are common in both adults and children in West Virginia, the development and evaluation of interventions to encourage safe riding practices, particularly those increasing helmet use, would be needed for both groups. More work in this area is greatly needed.

### Limitations

This study contributed to the limited extant literature regarding the relationship between changes in state-level off-road vehicle policies and the number of ATV/UTV-related trauma admissions in a state with the greatest burden of these types of injuries. Despite these strengths, this study is not without limitation. First, this study was conducted using patients treated at one of two Level I trauma centers located in West Virginia. In fact, these rates may be an underestimation, as the other Level I trauma center (Charleston Area Medical Center, Charleston, West Virginia) is located closer to a nationally recognized area in West Virginia known for ATV and UTV riding (Hatfield and McCoy Trails). This study did not investigate injuries incurred by individuals who died on site, were not transported to the medical facility, or injuries that did not receive any formal treatment. Secondly, this study did not compare its findings to a control location, as the authors did not have access to another state's ATV and UTV injury data. However, given the disparate number of ATV/UTV injuries that occur in West Virginia annually, it is not known if a truly suitable control state exists. Thirdly, there may be other confounding variables that were unaccounted for in the models. For example, during this time, the SARS-CoV-2 pandemic occurred. It is possible injuries may have spiked because of an increase in individuals’ free time created by lockdowns and Stay-at-home orders and not because of the Senate Bill 690 per se. However, research in West Virginia has shown that motor vehicle-related injuries resumed to normal levels quickly after stay-at-home orders issued during the pandemic were lifted.^42^ Lastly, at least two models were adjusted for the number of patrol officers employed within the state. It is possible that officers were not actually enforcing these laws during this time, given concerns regarding the spread of the SARS-CoV-2 virus and the social unrest regarding policing that was occurring nationwide during this time frame.^43^ Studies conducted in other states have shown that ATV laws are often sporadically enforced.^44^

## CONCLUSIONS

This study found the rate of ATV and UTV-related trauma admissions significantly increased after the passage of Senate Bill 690 in West Virginia. Given that ATV and UTV injuries are already disproportionately high in West Virginia compared to other states, these findings may suggest additional intervention or policy changes are needed to limit morbidity and mortality associated with these types of injuries. These findings may help guide other states that are interested in changing their ATV/UTV laws.
